# Recurrence Rate of Pulmonary Tuberculosis in Patients Treated with the Standard 6-Month Regimen: Findings and Implications from a Prospective Observational Multicenter Study

**DOI:** 10.3390/tropicalmed8020110

**Published:** 2023-02-10

**Authors:** Mohammed Saif Anaam, Alian A. Alrasheedy

**Affiliations:** 1 Department of Pharmacy Practice, Unaizah College of Pharmacy, Qassim University, Qassim 51911, Saudi Arabia; 2 Department of Pharmacy Practice, College of Pharmacy, Qassim University, Buraidah 51452, Saudi Arabia

**Keywords:** tuberculosis, TB, recurrent tuberculosis, relapse rate

## Abstract

Tuberculosis (TB) recurrence following successful treatment is a significant challenge in TB control programs. However, the rate of TB recurrence varies among studies. It depends on several factors, including the country/region where the investigation occurs, the study design, sample characteristics, and the anti-TB regimen used. In Yemen, a few previous studies examined the rate of TB recurrence and reported high recurrence rates, with a 5-year recurrence rate of approximately 9.5%. However, they were conducted before 2010 using the previous anti-TB regimen which was phased out and replaced with the World Health Organization’s (WHO) standard 6-month TB regimen. Consequently, this study aimed to examine the rate of TB recurrence after the implementation of the WHO standard 6-month regimen in Yemen. A prospective observational study was conducted with patients diagnosed with drug-susceptible pulmonary TB. The patients were recruited from five health centers with TB units in five governorates from January to December 2011. All the patients were followed up for five years after treatment completion. A total of 439 patients who completed the anti-TB regimen met the inclusion criteria and were included in the study. During the 5-year follow-up period, 8 patients (1.8%) died, and 13 patients (2.96%) were lost to follow-up, resulting in a final cohort of 418 patients. Of the cohort, 50.5% (n = 211) were male, while 49.5% (n = 207) were female patients. Of the patients, 129 patients (30.9%) were illiterate, 56 (13.4%) had cavitary pulmonary disease, and 6.2% (n = 26) had diabetes. The overall 5-year rate of TB recurrence in this study for the patients receiving the standard 6-month regimen was 2.9% (12/418). Moreover, almost half of the recurrent cases (41.7%; n = 5) were seen during the first year of the follow-up period. Some patient groups with risk factors recorded a higher recurrence rate, including patients with diabetes (15.4%), non-compliant patients (14.3%), pre-treatment lung cavitation patients (8.9%), illiterate patients (7.8%), and underweight patients (5.1%). In conclusion, the overall TB recurrence rate with the standard 6-month regimen was lower than that with the previous TB regimens. However, more efforts are needed to decrease TB recurrence rates further and achieve a durable cure for TB. In addition, healthcare professionals and TB control programs should consider potential risk factors of recurrence and address them to provide optimal care.

## 1. Introduction

Tuberculosis (TB) is a bacterial infectious disease caused by *Mycobacterium tuberculosis*. It is one of the leading causes of death globally [1,2]. TB is present in all countries with a varying rate of prevalence. In 2021, it was estimated that approximately 10.6 million individuals were infected with TB. This represented a 4.5% increase from 2020, during which 10.1 million individuals were infected with TB. The number of deaths from TB was estimated to be 1.6 million worldwide in 2021 [3,4]. In Yemen, TB is currently a primary infectious disease [5,6]. The incidence of TB has increased significantly in recent years, from 16.3 per 100,000 people in 2006 to 31.9 per 100,000 in 2018. This put an additional burden on the already strained healthcare system in Yemen. Several factors contributed to the increasing trend, including a low socioeconomic status, a weak healthcare system, political conflicts, and economic crises [6]. 

In 1970, the National Tuberculosis Control Program (NTCP) was established in Yemen to implement strategies and policies related to the control, treatment, and prevention of tuberculosis, including the World Health Organization (WHO) End Tuberculosis Strategy. In addition, the NTCP has adopted the directly observed therapy (DOT) Strategy since 1995 and expanded it gradually until full coverage in all the TB units in Yemen was realized [7]. 

The current WHO guidelines for the treatment of drug-susceptible pulmonary TB have recommended a 6-month regimen comprising four first-line anti-TB drugs in the intensive phase (for 2 months) and two drugs in the continuation phase (for 4 months): 2 months of isoniazid/rifampicin/pyrazinamide/ethambutol and 4 months of isoniazid/rifampicin (2HRZE/4HR) [8,9,10]. In addition, a daily dosing frequency for newly diagnosed patients with pulmonary TB is recommended in both phases of TB therapy (i.e., the intensive and continuation phases). This regimen is reported to have approximately a 85% success rate in treatment outcomes for TB patients [9]. In fact, this 6-month regimen, which uses rifampicin in both phases, i.e., for 6 months, was introduced in 2010 as it was shown to lead to better therapeutic outcomes and lower relapse rates compared to the 8-month regimen with 2-month administration of rifampicin (i.e., 2HRZE/6HE), which was phased out in 2010 [10,11,12]. 

The recurrence of TB following successful treatment is considered one of the main challenges posed to TB control in many countries. It is evident that even after successfully treating this infectious disease, individuals with a previous history of TB infection are at a higher risk of recurrent TB [13,14,15,16,17,18,19,20]. Moreover, the rate of TB recurrence differs widely among countries and regions [13]. A meta-analysis showed that the pooled incidence rate of recurrent TB was 1.47 per 100 person-years in low-TB-burden settings compared to 4.10 per 100 person-years in high-TB-burden settings [16]. In addition, the reported recurrence rate varies among studies in the literature. Many factors contribute to the variances in the rate of recurrent TB. These include the differences in study designs and characteristics (e.g., sample size, duration of follow-up) [13,16,21], the definition of TB recurrence in the studies [13,21,22], the TB burden in the country/region (high versus low prevalence of TB) [13,14,16], the study population and risk factors (e.g., comorbidities, HIV, diabetes, sociodemographic factors, smoking status) [21,23], the clinical characteristics of TB (e.g., presence of pre-treatment pulmonary cavities, sputum positivity after 2 months of treatment) [15,21,24], an anti-Tb drug regimen [16,17,25], and poor adherence to anti-tuberculosis drugs [17,23]. 

TB recurrence could cause higher morbidity and mortality, the development of drug resistance, the transmission of the infection to others, and further burden on healthcare systems [26]. Therefore, TB treatment aims at a durable cure for TB (i.e., without recurrence of TB) [17]. Consequently, it is essential to examine the recurrence rate of TB to provide guidance and further data for national TB control. This would help in designing plans and strategies to minimize recurrent TB. In Yemen, a few previous studies examined the rate of TB recurrence and reported high recurrence rates, with a 1-year recurrence rate of 5.7% [27] and a 5-year recurrence rate of 9.5% [25]. However, these studies were conducted before 2010 with the old drug regimen, i.e., the 8-month regimen with the 2-month administration of rifampicin only (2HRZE/6HE). Because of the high rates of treatment failures and relapses, this tuberculosis treatment was phased out in 2010 and replaced with the WHO standard 6-month regimen [11,12,25,28]. Consequently, this study aimed to examine the rate of TB recurrence after implementing the WHO standard regimen in Yemen. 

## 2. Methods

### 2.1. The Study Design, Setting, and Population

This prospective observational study was conducted with patients diagnosed with drug-susceptible pulmonary TB. In Yemen, similar to many low- and middle-income countries (LMICs), TB diagnosis is made by sputum smear microscopy for acid-fast bacilli (AFB), clinical manifestations and symptoms (i.e., clinical diagnosis), and other investigations (e.g., chest X-ray) [29,30,31]. The patients were recruited from January to December 2011, with the last enrolled patient completing the treatment on 30 June 2012. All the patients enrolled in this study were actively followed up for five years after treatment completion. The follow-up for the last enrolled patient was concluded in July 2017. The patients were recruited from five health centers with TB units in five governorates in Yemen: Al-Hodeida, Amran, Mareb, Ibb, and Taiz.

### 2.2. Anti-TB Regimen and Patient Follow-Up

The patients were treated using the WHO standard 6-month regimen that included 2 months of isoniazid/rifampicin/pyrazinamide/ethambutol and 4 months of isoniazid/rifampicin (2HRZE/4HR). The directly observed therapy (DOT) strategy was followed in the intensive phase of treatment, while community volunteers were involved in monitoring the patients in the continuous phase. During the initial and follow-up visits, the patients were told to report any symptoms they developed that may indicate the relapse of the disease to their TB unit. Consequently, when the patients returned to their TB unit with any symptoms associated with TB, a chest X-ray was performed, and a sputum acid-fast bacilli smear test was conducted when TB relapse was suspected [25]. 

### 2.3. Inclusion and Exclusion Criteria

The inclusion criteria in this study were as follows:

1. Patients diagnosed with pulmonary TB (smear-positive pulmonary TB).
2. Patients ≥ 15 years old.
3. Patients treated with the WHO standard 6-month regimen comprising 2 months of isoniazid/rifampicin/pyrazinamide/ethambutol and 4 months of isoniazid/rifampicin (2HRZE/4HR).
4. Patients whose 2-month smear conversion results were available.
5. Patients with complete follow-up data for 5 years after treatment completion.

The exclusion criteria included smear-negative pulmonary TB, other forms of TB, i.e., extra-pulmonary TB, patients ≤ 15 years old, and patients treated with other anti-TB regimens.

### 2.4. Study Main Outcome and Variables

The main outcome studied was the rate of TB recurrence. This was determined after one year and five years from treatment completion. TB recurrence was defined according to the WHO definition as “a patient previously treated for TB who has been declared cured or treatment completed and is diagnosed with bacteriologically positive TB (by sputum smear microscopy or culture)” [32]. 

Other independent variables collected in this study included sociodemographic data (e.g., age, gender, literacy status, marital status), clinical data (e.g., diabetes, cavitary pulmonary disease, body mass index (BMI), weight) and other data (e.g., smoking status, compliance). In this study, patients were considered adherent to TB therapy when they took ≥80% of the total prescribed doses of medicines in both the intensive and continuous phases of treatment [33]. In addition, patients who did not take their anti-TB drugs for ≥2 consecutive weeks were considered non-compliant with the DOT [34]. Cavitation or cavitary lung disease is the presence of cavities in a patient’s lungs due to TB disease. Cavitation can be present as a single cavity or multiple [35,36]. The cavities can vary widely in terms of their size and can have both thin and thick walls [37]. In this study, underweight patients had less than 90% of their ideal body weight at diagnosis [38].

### 2.5. Data Management and Statistical Analysis

The data analysis was performed using the IBM SPSS Statistics version 20.0 (IBM Corp., Armonk, NY, USA). The data were summarized using descriptive statistics, namely, frequencies, percentages, mean (M) with standard deviation (SD), and median with interquartile range (IQR). In addition, inferential statistics, namely chi-square and Fisher’s exact test, were used to examine the association between the study variables. Fisher’s exact test was used if more than 20% of the expected cell counts in the contingency table were less than 5 [39,40]. The statistical significance was set at a *p*-value < 0.05.

## 3. Results

### 3.1. Sociodemographic and Clinical Data of the Study Cohort

A total of 439 patients with pulmonary TB who completed the standard 6-month anti-TB regimen (2HRZE/4HR) met the inclusion criteria. They were subsequently included in the study. During the 5-year follow-up period, 8 patients (1.8%) died, and 13 patients (2.96%) were lost to follow-up, resulting in a final cohort of 418 patients. 

In this study, as shown in Table 1, 50.5% (n = 211) of the patients were male, while 49.5% (n = 207) were female. The mean age of the patients was 31 (SD = 14). A total of 177 patients (42.3%) were underweight. The mean BMI of the patients was 19.1 (SD = 1.9). Of the cohort, 129 patients (30.9%) were illiterate, 214 (51.2%) were not married, and 124 (29.7%) were smokers. In this study, 56 (13.4%) patients had cavitary pulmonary disease. The prevalence of diabetes in the cohort was 6.2% (n = 26). In terms of adherence, most patients had good adherence (≥80%) to their TB treatment regimen in both phases, while 28 (6.7%) patients were recorded as non-adherent to their anti-TB regimen. In this study, 97.1% (n = 406) of the patients had negative acid-fast bacilli (AFB) smears at the end of the two months. The results are summarized in Table 1. 

### 3.2. Overall Recurrence Rate of TB 

**Figure 1:**
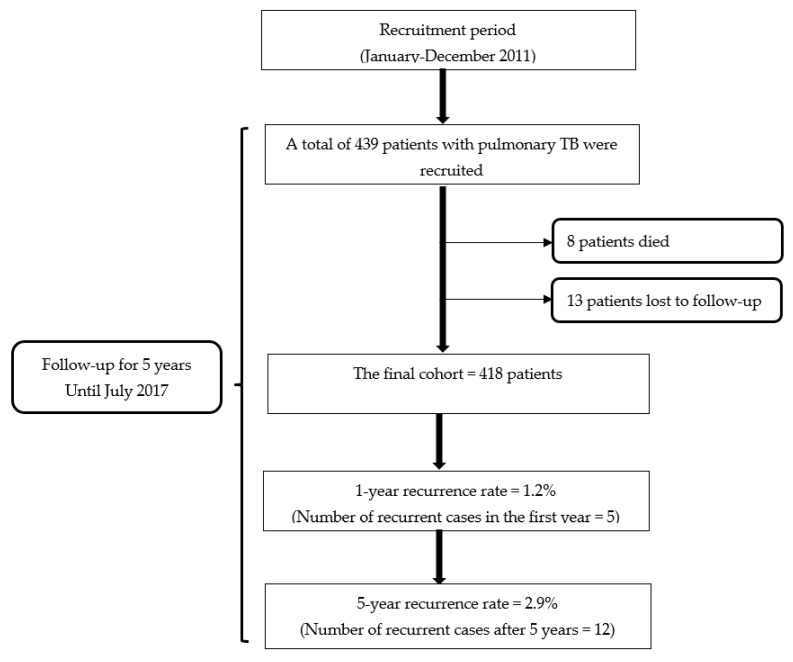
Flowchart of the study.

The overall 5-year rate of TB recurrence in this study for the patients receiving the WHO standard 6-month regimen was 2.9% (12/418). Moreover, it was noted that almost half of the recurrent cases (41.7%; n = 5) were seen during the first year of the follow-up period. This indicated that the rate of TB recurrence in the first year was 1.2% (Figure 1). In this study, 11 recurrent cases were identified among the patients whose treatment outcome was declared cured (n = 406) and 1 case among the patients whose treatment outcome was treatment completion (n = 12). 

### 3.3. Recurrence Rate of TB according to Patient’s Subgroups and Regions 

We examined the recurrence rate among 12 subgroups of patients according to the presence of specific risk factors. The recurrence rate was significantly higher in eight subgroups (Table 2). Some patient groups with risk factors recorded a higher recurrence rate, including underweight patients, illiterate patients, pre-treatment lung cavitation, non-complaint patients, and patients with diabetes. Of the underweight patients, 5.1% had a recurrence of TB compared to 1.2% of patients with no malnutrition (*p* = 0.020). A higher proportion of illiterate patients (7.8%) had recurrent TB compared to literate patients (0.7%) (*p* < 0.001). Patients with lung cavitation had a recurrence rate of 8.9% compared to those without cavitation (1.9%) (*p* = 0.014). Similarly, patients who were non-compliant with anti-TB drug therapy had a higher recurrence rate than compliant patients (14.3% and 2.1%, respectively, *p* = 0.006). In this study, patients with diabetes had a higher recurrence rate than non-diabetic patients (15.4% and 2.0%, respectively, *p* = 0.004). In addition, we examined the TB recurrence rate among the patients according to their geographic region. However, there were no statistically significant differences among the five governorates (*p* = 0.109). The results are summarized in Table 2.

## 4. Discussion

This is the first study from Yemen that examined the rate of TB recurrence after the completion of the WHO standard 6-month regimen. The 5-year rate of TB recurrence in this study was 2.9%. This is lower than the recurrence rate seen with the 8-month regimen, which was reported previously to be 9.5% [25]. This finding aligns with previous studies that reported a lower recurrence rate when using the standard 6-month regimen [41,42]. In fact, a meta-analysis showed that the pooled relapse rate with the 8-month regimen with 1–2 months of rifampicin was 16% compared to 3.8% for regimens including 6–7 months of rifampicin. The adjusted incidence rate ratio for relapse was 3.6 (95% CI = 2.5 to 5.3) higher than the regimen including 6 months of rifampicin [42]. Moreover, recent findings from a meta-analysis by Vega et al. 2021 show that a TB regimen with less than 6 months of rifampicin had a higher rate of recurrence than a regimen of 6 or more months (adjusted incidence rate ratio (95% CI) = 1.61 (1.05 to 2.47) [16]. 

The TB recurrence rate in this study was higher than in some studies. Zhisong et al. (2021) reported a 10-year recurrence rate (2010–2020) of 1.2% in Fujian, China [43]. In addition, the overall recurrence rate in a study conducted in Henan Province, China, from 2005 to 2018 was 1.5% [22]. Similarly, in a study from Singapore, the recurrence rate was 1.2% (n = 91) among the 7478 cases recorded during 2006–2013 [17]. In a study from Barcelona, Spain, a recurrence rate of 1.3% was reported for patients who completed treatment between 2003 and 2006 and were followed up until December 2009 [44]. In another study, which analyzed the database notifications from a large center in Leicester, UK, between 1994 and 2014, a recurrence rate of 1.8% was reported [45]. 

However, the TB recurrence rate in this study was lower than the recurrence rate reported in some other studies in the literature. Youn et al. (2022) reported a recurrence rate of 6.7% in Korea in a nationwide cohort study during 2002–2013 [13]. Ruan et al. 2022 reported a 5-year recurrence rate of 5.4% in Hangzhou, China [15]. In a retrospective cohort study of successfully treated TB patients during 2009–2020 in Sichuan Province, China, Li et al. 2022 reported a recurrence rate of 4.9% [46]. However, in a prospective longitudinal study in Jiangxi Province, China, Lin et al. 2021 reported a 7-year recurrence rate of 15.2% [21]. A recurrence rate of 4.88% was reported by a retrospective observational study from Carapicuíba, Brazil, that included a cohort of patients during 2000–2010 with follow-ups conducted till the end of 2012 [47]. A study from Cape Town, South Africa, reported that 8% of TB patients who completed treatment had recurrent TB over 13 years (2003–2016) [14]. 

As mentioned earlier, it is essential to emphasize that several factors could contribute to the differences in recurrence rates among studies. These include the study design (prospective versus retrospective), duration of follow-up (e.g., 5, 7, and 10 years), TB regimen (e.g., drugs used in the regimen, dosing schedules, duration), studied population (e.g., general population, HIV population), the definition of recurrence, burden of TB in the country/region (low, moderate, high prevalence of TB), majority of risk factors for recurrence in the study samples, and level of adherence to the TB regimen. All these factors could explain the differences among studies. Consequently, the head-to-head comparisons among studies should be interpreted considering these factors [22]. 

This study noted that almost half of the recurrences (41.7%) occurred in the first year after treatment completion. This confirms the findings of previous studies, which reported that most recurrences occur in the first 1–2 years from treatment completion. Ruan et al., in their 2022 study, indicated that more than half of TB recurrences (51.1%) occurred within one year of treatment completion [15]. Furthermore, it was reported that most recurrences (89%) happened within the first 2 years [13]. Another study showed that more than half of the recurrence cases (55.2%) occurred in the first 2 years after treatment completion [21]. Another study showed that most recurrences of TB (74.4%) occurred within three years [46]. Consequently, our findings, along with the conclusions of the literature, provide further guidance and highlight the need to follow-up and monitor successfully treated TB patients, especially during the first 1–2 years, when most recurrences occur. This will help provide suitable early treatment for recurrent cases, preventing TB complications and disease transmission to others in the community. In addition, it was noted that the recurrence rate was higher in subgroups of patients with potential risk factors. These included patients with diabetes (15.4%), patients with a lack of adherence (14.3%), patients with pre-treatment lung cavitation (8.9%), illiterate patients (7.8%), and underweight patients (5.1%). These findings are consistent with the widely reported risk factors in the literature, including diabetes [48,49,50,51,52], non-adherence [25,53], lung cavitation [15,24,36,54,55,56], and underweight patients [13,56,57,58]. Consequently, healthcare professionals and TB control programs must consider these risk factors, which may hinder successful TB treatment, and provide appropriate support and interventions. In addition, future research should further examine the risk factors for the recurrence of TB and develop interventions, including educational, therapeutic, and other interventions, to address them and provide optimal care. 

The study had several implications. Overall, it is encouraging that the recurrence rate was lower after implementing the standard 6-month regimen in Yemen. This would help control TB and lower its burden on the patients and the healthcare system. Moreover, the current study’s findings indicated that most recurrent episodes occurred during the early years after treatment completion (i.e., 41.7% within the first year). Consequently, based on this finding and other recent studies in literature, TB control programs need to monitor TB patients in the first years following the completion of their treatment. Furthermore, TB patient care should go beyond treatment completion to include a follow-up and monitoring of the patients as part of the TB control strategy [21,59]. This is especially important for patients with a higher risk of recurrence. In addition, healthcare professionals must address modifiable factors that could increase the risk of TB recurrence. These include addressing the non-adherence, which was noted as relatively high in this study (6.7%). Moreover, integrated care that ensures the appropriate management of diabetes (i.e., glycemic control) should be implemented as part of the TB control program in coordination with other healthcare professionals. Quality TB care should go beyond the focus only on monitoring strategies. In particular, financial, social, and psychological support should be provided to patients from low socioeconomic status and with low literacy status [60,61]. This could improve patient care, and address the current challenges, such as poor compliance and malnutrition, and improve patients’ awareness of TB management. This is particularly important in Yemen, given the high level of poverty (approximately 50% of the population below the poverty line), fragile healthcare system, widespread food insecurity and malnutrition, shortages of energy and water supply, weak infrastructure, and issues related to access to healthcare services [62,63]. Future studies should address the gaps in the care provided to TB patients, especially the post-treatment recurrence of TB. The studies could examine the best strategies and interventions to prevent recurrence and the effective strategies that could be incorporated into routine TB control programs to identify recurrent TB cases early. 

## 5. Study Strengths and Limitations

This study had some strengths. This prospective study had a relatively long follow-up period (i.e., a 5-year follow-up after treatment completion). It was a multicenter study, and the patients were recruited from five centers in five governorates in Yemen. However, the study had some limitations. As the number of recurrence cases was small, it was impossible to proceed with multivariable logistic regression to quantify the risk factors further. This is because it might not have been reliable, given that the number of events (i.e., cases) in some variables (i.e., predictors) was less than 10. This would have affected the accuracy and precision of the regression coefficient of the independent variable [64,65,66]. However, these risk factors are consistent with the literature and widely reported in previous studies, as discussed earlier. As our study focused on the overall rate of TB recurrence, future studies could include larger samples and focus on specific risk factors to provide further evidence. In addition, as the proportion of patients recruited from some governorates is relatively small, future studies with larger samples from each governorate could be conducted to examine whether there is a difference in the recurrence rate among the different governorates in Yemen. However, overall, we believe our study findings give more guidance and valuable data that can help in the control and management of TB. 

## 6. Conclusions

The overall TB recurrence with the standard 6-month regimen in Yemen was relatively low compared to previous TB regimens. However, further efforts are needed to decrease TB recurrences and achieve a durable cure for TB. In addition, healthcare professionals and TB control programs need to consider the potential risk factors of recurrence and address them to provide optimal care. 

## Figures and Tables

**Table 1:** Sociodemographic and clinical data of the patients (n = 418).

Characteristics	N (%) Unless Otherwise Specified
Age (in years) Mean ± SD Median (IQR)	31 ± 14 25 (21–40)
Gender	
Male	211 (50.5%)
Female	207 (49.5%)
Literacy status	
Literate	289 (69.1%)
Illiterate	129 (30.9%)
Marital status	
Married	204 (48.8%)
Non-married	214 (51.2%)
Smoking status	
Smokers	124 (29.7%)
Non-smokers	294 (70.3%)
Presence of cavitary lung disease	
Yes	56 (13.4%)
No	362 (86.6%)
Results of acid-fast bacilli (AFB) smear at diagnosis	
1+ positive	297 (71.1%)
2+ positive	76 (18.2%)
3+ positive	45 (10.8%)
Results of acid-fast bacilli (AFB) smear at the end of two months	
Positive	12 (2.9%)
Negative	406 (97.1%)
Weight gain ≤5% *	
Yes	216 (51.7%)
No	202 (48.3%)
Underweight	
Yes	177 (42.3%)
No	241 (57.7%)
BMI (kg/m^2^) Mean (SD) Median (IQR)	19.1 ± 1.9 19.1 (18.3–19.8)
Compliance	
Yes	390 (93.3%)
No	28 (6.7%)
Presence of diabetes	
Yes	26 (6.2%)
No	392 (93.8%)

* Weight gain during the intensive phase.

**Table 2:** Overall, 5-year rate of TB recurrence in the cohort and rates of recurrence in patients stratified by groups.

Variable	Recurrence n (%)	*p*-Value ^a^
Overall rate (n = 418)	12 (2.9)	-
Age		
Age ≥ 45 years (n = 84)	3 (3.6%)	0.714 *
Age < 45 (n = 334)	9 (2.7%)	
Gender		
Female (n = 207)	10 (4.8%)	0.017
Male (n = 211)	2 (0.9%)	
Literacy status		
Literate (n = 289)	2 (0.7%)	<0.001 *
Illiterate (n = 129)	10 (7.8%)	
Marital status		
Married (n = 204)	2 (1.0%)	0.024
Non-married (n = 214)	10 (4.7%)	
Smoking status		
Non-smoker (294)	6 (2.0%)	0.195 *
Smokers (n = 124)	6 (4.8%)	
Presence of cavitary lung disease		
Yes (n = 56)	5 (8.9%)	0.014 *
No (n = 362)	7 (1.9%)	
Results of acid-fast bacilli (AFB) smear at the diagnosis		
1+ positive (n = 297)	6 (2.0%)	0.114 *
2+/3+ positive (n = 121)	6 (5.0%)	
Results of acid-fast bacilli smear at the end of two months		
Positive (n = 12)	1 (8.3%)	0.298 *
Negative (n = 406)	11 (2.7%)	
Weight gain ≤ 5%		
Yes (n = 216)	10 (4.6%)	0.026
No (n = 202)	2 (1.0)	
Underweight		
Yes (n = 177)	9 (5.1%)	0.020
No (n = 241)	3 (1.2%)	
Compliance		
Yes (n = 390)	8 (2.1%)	0.006 *
No (n = 28)	4 (14.3%)	
Presence of diabetes		
Yes (n = 26)	4 (15.4%)	0.004 *
No (n = 392)	8 (2.0%)	
Governorate		
Al-Hodeida (n = 165)	5 (3.0%)	0.109 *
Taiz (n = 125)	4 (3.2%)	
Ibb (n = 87)	0 (0%)	
Mareb (n = 25)	2 (8%)	
Amran (n = 16)	1 (6.3%)	

^a^*p* value based on chi-square test and Fisher’s exact test. * Indicates Fisher’s exact test.

## Data Availability

The data that support the findings of this study are available from the corresponding author upon reasonable request.
